# Rapid disease progression in a patient with mismatch repair-deficient and cortisol secreting adrenocortical carcinoma treated with pembrolizumab

**DOI:** 10.1053/j.seminoncol.2018.06.001

**Published:** 2018-06

**Authors:** R.T. Casey, O. Giger, I. Seetho, A. Marker, D. Pitfield, L.H. Boyle, M. Gurnell, A. Shaw, M. Tischkowitz, E.R. Maher, V.K. Chatterjee, T. Janowitz, G. Mells, P. Corrie, B.G. Challis

**Affiliations:** a Department of Endocrinology and Diabetes, Cambridge University NHS Foundation Trust, Cambridge, UK; b Department of Medical Genetics, Cambridge University, Cambridge, UK; c Department of Histopathology, Cambridge University NHS Foundation Trust, Cambridge, UK; d Department of Pathology, University of Cambridge, Cambridge, UK; e Department of Radiology, Cambridge University NHS Foundation Trust, Cambridge, UK; f Department of Medical Oncology, Cambridge University NHS Foundation Trust, Cambridge, UK; g Department of Hepatology, Cambridge University NHS Foundation Trust, Cambridge, UK; h IMED Biotech Unit, Clinical Discovery Unit, AstraZeneca, Cambridge, UK

**Keywords:** Adrenocortical carcinoma, Lynch syndrome, Cortisol, Pembrolizumab, Hepatitis

## Abstract

**Context:**

Metastatic adrenocortical carcinoma (ACC) is an aggressive malignancy with a poor prognosis and limited therapeutic options. A subset of ACC is due to Lynch syndrome, an inherited tumor syndrome resulting from germline mutations in mismatch repair (MMR) genes. It has been demonstrated that several cancers characterized by MMR deficiency are sensitive to immune checkpoint inhibitors that target PD-1. Here, we provide the first report of PD-1 blockade with pembrolizumab in a patient with Lynch syndrome and progressive cortisol-secreting metastatic ACC.

**Case report:**

A 58-year-old female with known Lynch syndrome presented with severe Cushing's syndrome and was diagnosed with a cortisol-secreting ACC. Three months following surgical resection and adjuvant mitotane therapy the patient developed metastatic disease and persistent hypercortisolemia. She commenced pembrolizumab, but her second cycle was delayed due to a transient transaminitis. Computed tomography performed after 12 weeks and 2 cycles of pembrolizumab administration revealed significant disease progression and treatment was discontinued. After 7 weeks, the patient became jaundiced and soon died due to fulminant liver failure.

**Conclusion:**

Treatment of MMR-deficient cortisol-secreting ACC with pembrolizumab may be ineffective due to supraphysiological levels of circulating corticosteroids, which may in turn mask severe drug-induced organ damage.

## Introduction

Adrenocortical carcinoma (ACC) is a rare and aggressive cancer with limited therapeutic options for patients with advanced disease. In adults, the majority of ACC are sporadic; however, it is recognized that a subset of ACC is associated with Lynch syndrome, an autosomal dominant tumor syndrome caused by pathogenic mutations of DNA mismatch repair (MMR) genes (*MSH2, MSH6, PMS2*, and *MLH1*) [1], [2]. MMR-deficient tumors possess a high somatic mutation burden and present a diverse repertoire of neoantigens which in turn enhances tumor immunogenicity and thus tumor-specific T cell responses. This is relevant in the context of the important advances in T cell immune checkpoint targeted cancer immunotherapy, in particular with drugs that target programmed death-ligand 1 (PD-L1). PD-L1 is expressed on the surface of tumor cells and inhibits T cell activation through binding of its cognate coinhibitory T cell receptor, PD-1. In many tumors, PD-L1 expression is upregulated, enabling tumor cells to evade the host immune system through inhibition of activated T cells. Immune checkpoint inhibitors that target this pathway, by blocking either PD-1 or PD-L1, have led to significant clinical responses in some patients with different cancer types [3]. Indeed, recent landmark studies have now demonstrated that MMR-deficient tumors are more responsive to PD-1 blockade with the anti-PD-1 antibody pembrolizumab than MMR-proficient tumors, with response rates exceeding 50% [4]. Based on these data, pembrolizumab was granted Food and Drug Administration approval for the treatment of unresectable or metastatic, microsatellite instability-high (MSI-H) or MMR-deficient solid tumors regardless of tumor site or histology. One of the limitations of T cell checkpoint targeted cancer immunotherapy are T cell mediated autoimmune side effects, that can be severe and life threatening, but can be ameliorated with immune suppressive medications, such as glucocorticoids [5]. This demonstrates that the enhanced T cell response may be sensitive to or prevented by the high levels of cortisol that are found in patients with cortisol secreting ACC.

Here, we describe the first reported case of a cortisol-secreting metastatic ACC treated with pembrolizumab in a patient with Lynch syndrome.

## Methods and materials

MMR immunohistochemistry was performed using the Ventana Benchmark MMR panel: MLH-1 (M1), PMS2 (EPR3947), MSH2 (G219-1129), and CONFIRM anti-MSH6. PD-L1 immunohistochemistry was performed using the PD-L1 IHC 22C3 pharmDx test (Dako) and β2-microglobulin immunohistochemistry was performed using a polyclonal rabbit antihuman antibody (Dako, Cat# A0072). Immunohistochemistry was performed on 4 µm sections of paraffin embedded tumor tissue, in accordance with the manufacturer's guidelines and interpreted by an experienced pathologist (OG).

## Results and clinical case description

**Fig. 1:**
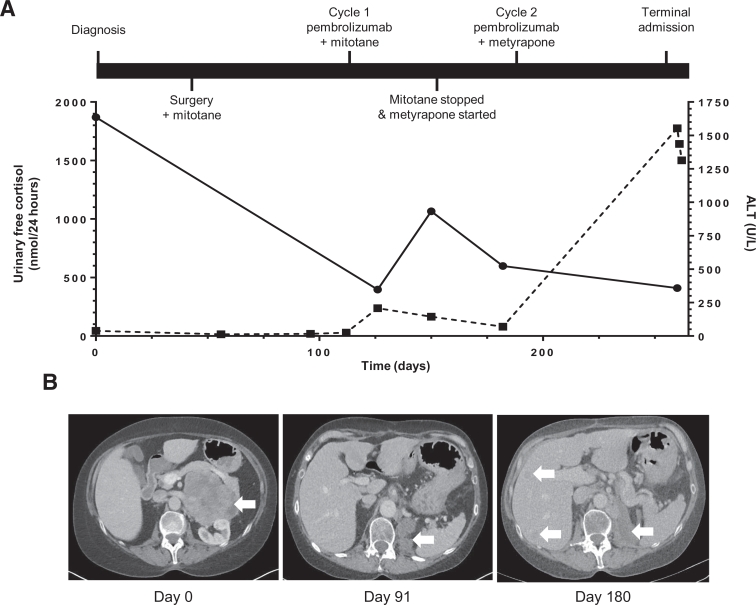
Time course of laboratory and radiological results. **(A)** The 24-hour urinary cortisol and ALT concentrations plotted against time (days). Solid line indicates urinary free cortisol levels. Dashed line indicates ALT levels. **(B)** Representative cross-sectional CT images at diagnosis (day 0), postadrenalectomy (day 91), and following initiation of pembrolizumab therapy (day 180). White arrowheads indicate the primary tumor at diagnosis and, following treatment, sites of local and metastatic recurrence.

A 58-year-old female presented with a 2 year history of progressive weight gain and reduced mobility. Her medical history was significant for Lynch syndrome due to a pathogenic mutation in *MSH2* (p.Asn263fs). The patient had previously undergone an abdominal hysterectomy and bilateral salpingo-oophorectomy following identification of cervical cell dysplasia. Annual colonoscopic examinations were normal. There was no history of glucocorticoid exposure. Clinically, the patient was hypertensive (165/130 mmHg) with an elevated body mass index (30 kg/m^2^). She was hirsute, profoundly plethoric with widespread ecchymoses and exhibited a marked proximal myopathy. Abdominal examination revealed violaceous abdominal striae, in the absence of any organomegaly or palpable masses. Biochemical investigations revealed marked autonomous adrenocorticotrophic hormone-independent hypercortisolemia (urinary free cortisol 1870 nmol/24 h [normal range: <146 nmol/24 h]; adrenocorticotrophic hormone < 5 ng/L (normal range: <50 ng/L)) (Fig. 1**A**) and hyperandrogenism (testosterone 4.8 nmol/L [normal range: 0.2–3 nmol/L]); androstenedione 22.7 nmol/L [normal range: 1.4–4.3 nmol/L]). Computed tomography (CT) demonstrated an 11 cm × 7 cm heterogeneous lesion arising from the left adrenal and no evidence of metastatic disease **(**Fig. 1**B)**.

**Fig. 2:**
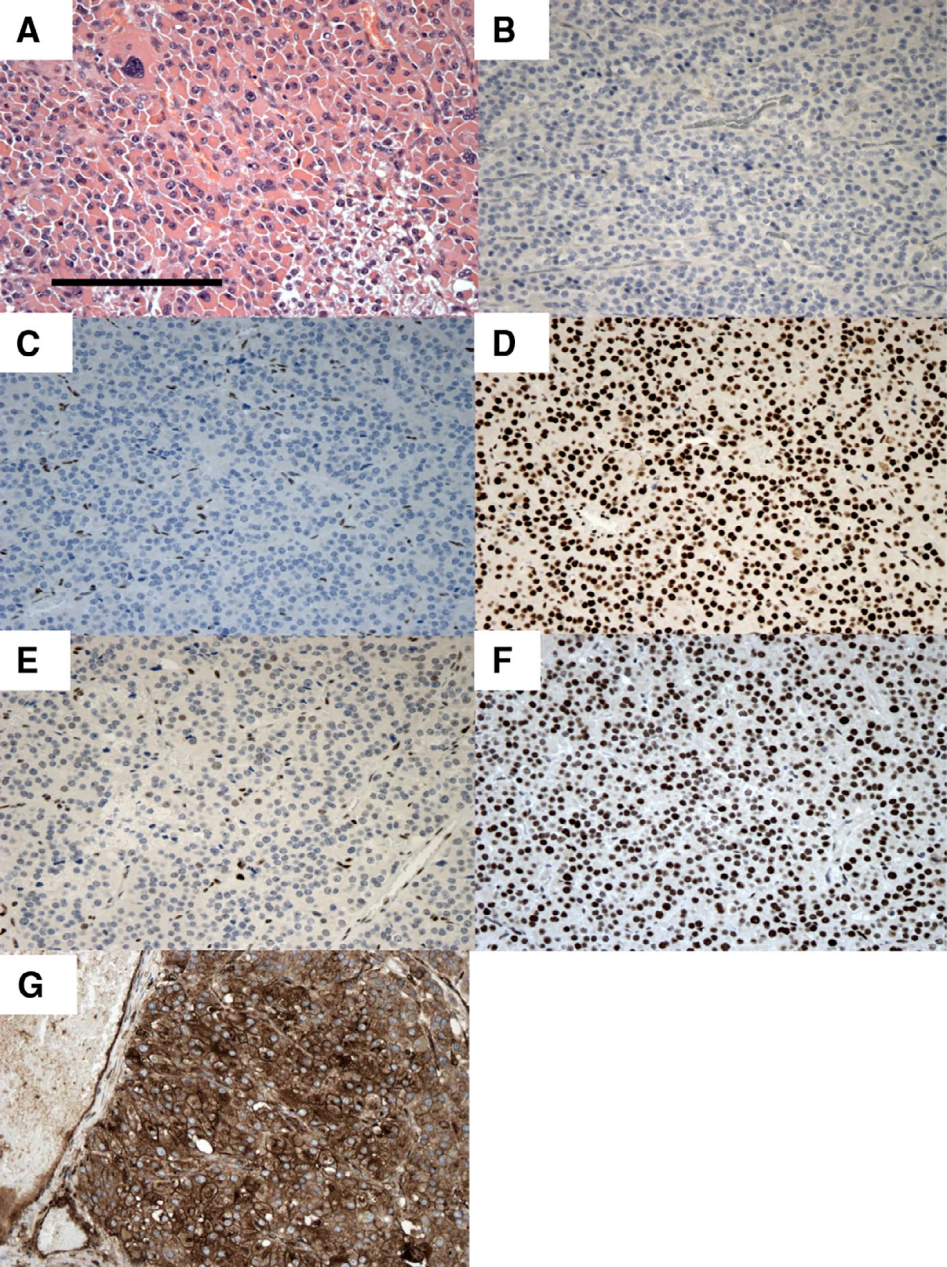
**(A)** Representative hematoxylin and eosin-stained photomicrograph of ACC. **(B–G)** Immunohistochemical analyses of protein expression in resected ACC. **(B)** PD-L1 expression in tumor cells was <1%. The tumor did not express **(C)** MSH2 or **(E)** MSH6 but expression of **(D)** MLH1, **(F)** PMS2, and **(G)** β2-microglobulin was preserved (scale bar = 200 µm).

The patient underwent an uncomplicated left adrenalectomy and nephrectomy. Subsequent pathological examination of the resected tumor confirmed a stage III ACC (modified Weiss Score 9). Few tumor infiltrating lymphocytes were identified and tumor PD-L1 expression was low (<1%) (Fig. 2**A and B)**. Immunohistochemical analysis of the tumor demonstrated an isolated loss of MSH2 and MSH6 expression with preserved expression of MLH1 and PMS2 **(**Fig. 2**C–F).** Adjuvant mitotane therapy (up to a maximum tolerated dose of 2,000 mg each day) was commenced with concomitant hydrocortisone replacement therapy (40 mg daily in divided doses).

Three months following surgery the patient developed worsening abdominal pain. CT revealed tumor recurrence in the left adrenal bed and new hepatic metastases **(**Fig. 1**B).** In view of disease recurrence in the context of a MMR-deficient tumor, the patient was commenced on pembrolizumab (2 mg/kg) in combination with mitotane (2,000 mg daily). Following the first cycle of pembrolizumab, the patient developed a mild elevation of serum alanine aminotransferase (peak ALT 208 U/L [normal range: 7–40 U/L]). Since the differential diagnosis for the elevation of serum ALT included mitotane-induced hepatotoxicity and immunotherapy-induced autoimmune hepatitis, mitotane was discontinued and the second cycle of pembrolizumab was delayed. Within 14 days of discontinuing mitotane, liver function had improved (ALT 129 U/L) and continued to do so when assessed 9 days later (ALT 70 U/L). During this period metyrapone was commenced at a dose of 2,000 mg per day; however, a 24 hour urinary cortisol rose to 1,066 nmol/24 h **(**Fig. 1**A)**. Metyrapone was further increased to 3,000 mg daily and the patient proceeded to receive a second cycle of pembrolizumab without any further disturbance in liver function tests, although treatment tolerance was poor, due to nausea, vomiting, and fatigue. Twelve weeks following initiation of treatment, CT imaging revealed significant disease progression, with both rapidly increased size of the relapsed disease at the primary site and multiple new liver metastases **(**Fig. 1**B**).

The clinical management was changed to focus on best supportive care and pembrolizumab treatment was, therefore, discontinued. Seven weeks later, the patient was admitted to hospital, grossly jaundiced (bilirubin 371 µmol/L [normal range: 0–20 µmol/L]). Biochemical indices of liver function were markedly deranged with ALT 1,553 U/L (normal range: 7–40 U/L); alkaline phosphatase 530 U/L (normal range: 30–130 U/L); and international normalized ratio (3.7; Fig. 1**A**). Liver ultrasound showed only limited increase in size of liver metastases with no new lesions identified. Viral hepatitis serology was negative. A liver biopsy was not feasible due to the presence of coagulopathy and rapid clinical deterioration. The possibility of pembrolizumab-induced autoimmune hepatitis could not be excluded, so methylprednisolone (2 mg/kg) and mycophenolate mofetil (500 mg twice daily) were commenced. However, the patient developed fulminant liver failure and died 3 days later. Both the family and coroner declined post mortem examination.

## Discussion

In 2017, the Food and Drug Administration granted accelerated approval for pembrolizumab use in any solid tumor shown to be MSI-high or MMR-deficient. This decision was based on clinical trial data demonstrating high response rates in patients with advanced cancer whose tumors had these genetic signatures [4], [6]. Most of the patients had received multiple preceding lines of treatment. In their most recent study, Le et al investigated the efficacy of pembrolizumab in patients with progressive MMR-deficient tumors across 12 different cancer types, and reported an objective response rate of 53% [4].

ACC was not represented in these initial studies, but is a recognized Lynch syndrome associated malignancy [1], [2]. Whether sporadic or associated with an inherited tumor syndrome, the standard treatment for patients with good performance status and unresectable or metastatic ACC is currently combination chemotherapy consisting of etoposide, doxorubicin, cisplatin, and mitotane. Unfortunately, this combination therapy is associated with dose - limiting side effects and serious adverse events, while only resulting in a modest improvement in median overall survival [7]. Therefore, there remains an unmet therapeutic need for patients with advanced ACC. That MMR-deficient cancers are sensitive to immune checkpoint blockade provides a potential precision medicine opportunity for the relevant subset of patients with ACC.

Immune checkpoint inhibitors are significantly improving clinical outcomes in some advanced cancers, although most patients progress with their disease despite treatment. Both primary and acquired resistance mechanisms have been described, and may be secondary to the intrinsic and extrinsic factors to tumor cells [3], [8], [9]. One of the cancer cell intrinsic immune escape mechanisms is impaired antigen presentation by cancer cells due to reduced expression of peptide-MHC processing components [3]. In our patient's primary tumor, we found preserved β2-microglobulin expression, with a distinct membranous staining pattern within the parenchyma (Fig. 2**G**). While this is not a comprehensive assessment of the antigen processing pathway, these data suggest that other mechanisms may account for the immunotherapy failure we observed. As such, the impact of systemic glucocorticoid exposure on immunotherapy responsiveness should be considered, but is incompletely understood [10]. The case we describe provides a rare opportunity to examine the relationship of systemic immune suppression with increased MMR-deficiency mediated cancer cell antigenicity in the context of cancer immunotherapy.

Glucocorticoids regulate many aspects of immune function, including suppression of the cellular Th1 immune responses that are ostensibly required for the antitumor effects of immune checkpoint inhibitors. Indeed, preclinical studies in a rodent model of pancreatic ductal adenocarcinoma demonstrated that elevated plasma corticosterone levels impaired immunotherapy outcome [11]. The resected ACC tumor in the case described here was characterized by a paucity of tumor infiltrating T lymphocytes. Typically, MMR-deficient tumors display an increased number of lymphocytes within the tumor which correlates with response to immune checkpoint inhibition and prognosis [3]. The discordance between these findings and our case may reflect the suppressive effects of glucocorticoids on T cell chemotaxis and migration, possibly potentiated by the high local concentration in the tumor microenvironment, and, at least in part, explains the poor response to immune checkpoint blockade. Glucocorticoids have also been shown to induce Fas-L overexpression in preclinical models of ACC, suggesting existence of additional glucocorticoid-regulated mechanisms that may impair response to immunotherapy [12]. Furthermore, our case demonstrates the potential for fluctuating levels of endogenous glucocorticoids to potentially influence response to immune-related adverse events.

The patient described in this report developed transient liver dysfunction after the first cycle of pembrolizumab. Immunotherapy with pembrolizumab was discontinued after cycle 2 due to rapid disease progression. The patient developed fatal liver failure that did not respond to treatment with immune suppression 7 weeks after stopping pembrolizumab. Hepatitis is a recognized immune-related adverse event associated with pembrolizumab, occurring in approximately 1% of all treated patients. Higher rates of liver damage have been reported when potentially hepatotoxic agents, such as dacarbazine, are given in combination with checkpoint inhibitors [13]. We speculate that concurrent treatment with the hepatotoxic agents, mitotane and metyrapone, may have increased the risk of liver injury, but cannot completely exclude the possibility that liver failure was due to disease progression. It is notable that conflicting data exist whether the occurrence of immune-related adverse events are associated with improved response to treatment [14]. This again could be reflective of the pretreatment state of T cell infiltration into tumor and normal tissue. In the case discussed here, it is conceivable that the very high intratumoral cortisol concentration may have caused a more profound intratumoral immune suppression when compared to the liver tissue. This could have reduced the therapeutic index and caused pembrolizumab toxicity in the absence of antitumor efficacy.

Glucocorticoids are the mainstay of treatment for immune-related adverse events, and to date, there is no evidence that their use once the effect of immunotherapy has been established impacts upon treatment outcomes. However, in this case glucocorticoids were elevated when treatment commenced, which represents a distinct clinical scenario. Our report highlights a need for further investigations to determine whether supraphysiological concentrations of glucocorticoid (endogenous or exogenous) induce a degree of systemic immunosuppression that impacts upon clinical response to immune checkpoint inhibition.

In summary, we report the first description of pembrolizumab treatment in a MMR-deficient and cortisol-secreting metastatic ACC. The therapy was not effective and rapid disease progression occurred. Although no single case should be used to argue against recommendations based on comprehensive scientific and clinical investigation, it is important to acknowledge potential immunosuppressive phenomena that may reduce the efficacy of cancer immunotherapy, and cases can serve as a salutary reminder that there can be exceptions to the rule.

## References

[1] Raymond VM, Everett JN, Furtado LV (2013). Adrenocortical carcinoma is a lynch syndrome-associated cancer. J Clin Oncol.

[2] Challis BG, Kandasamy N, Powlson AS (2016). Familial adrenocortical carcinoma in association with lynch syndrome. J Clin Endocrinol Metab.

[3] Chen DS, Mellman I (2017). Elements of cancer immunity and the cancer-immune set point. Nature.

[4] Le DT, Durham JN, Smith KN (2017). Mismatch repair deficiency predicts response of solid tumors to PD-1 blockade. Science.

[5] Weber JS, Yang JC, Atkins MB, Disis ML (2015). Toxicities of Immunotherapy for the practitioner. J Clin Oncol.

[6] Le DT, Uram JN, Wang H (2015). PD-1 blockade in tumors with mismatch-repair deficiency. N Engl J Med.

[7] Fassnacht M, Terzolo M, Allolio B (2012). Combination chemotherapy in advanced adrenocortical carcinoma. N Engl J Med.

[8] Jenkins RW, Barbie DA, Flaherty KT (2018). Mechanisms of resistance to immune checkpoint inhibitors. Br J Cancer.

[9] Flint TR, Fearon DT, Janowitz T (2017). Connecting the metabolic and immune responses to cancer. Trends Mol Med.

[10] Connell CM, Raby SEM, Beh I (2018). Cancer immunotherapy trials underutilize immune response monitoring. Oncologist.

[11] Flint TR, Janowitz T, Connell CM (2016). Tumor-induced IL-6 reprograms host metabolism to suppress anti-tumor immunity. Cell Metab.

[12] Wolkersdorfer GW, Marx C, Lohmann T (1998). The mercy of adrenocortical tumor cells on lymphocytes. Endocr Res.

[13] Robert C, Thomas L, Bondarenko I (2011). Ipilimumab plus dacarbazine for previously untreated metastatic melanoma. N Engl J Med.

[14] Postow MA, Sidlow R, Hellmann MD (2018). Immune-related adverse events associated with immune checkpoint blockade. N Engl J Med.

